# Pharmacological mechanisms and potential clinical applications of Dihydromyricetin in neurological disorders

**DOI:** 10.3389/fphar.2025.1618623

**Published:** 2025-07-16

**Authors:** Yike Zhang, Tingting Zhang, Manli Zhao, Peichun Li, Tao Liu, Jiangbo Xie

**Affiliations:** ^1^ Clinical Integration of Traditional Chinese and Western Medicine, Shandong University of Traditional Chinese Medicine, Jinan, China; ^2^ Department of Neurology, Weifang Traditional Chinese Hospital, Weifang, China

**Keywords:** Dihydromyricetin, central nervous system, pharmacological effects, Alzheimer’s disease, Parkinson’s disease

## Abstract

Neurological disorders (e.g., Alzheimer’s disease, Parkinson’s disease, and stroke) have complex pathogenesis and affect a substantial proportion of the population; yet, available treatments have poor or limited efficacy, and the patients have a poor prognosis, with high morbidity and mortality. Dihydromyricetin (DHM), a flavonoid compound extracted from plants, has received widespread attention in recent years because of its diverse pharmacological effects. *In vitro* and *in vivo* studies have revealed its substantial antioxidant, anti-inflammatory, and neuroprotective properties, making it a promising candidate for the treatment of central nervous system disorders through multiple mechanisms and pleiotropic effects. Therefore, there is an urgent need to develop novel therapeutic strategies. DHM is an attractive candidate for the management of neurological disorders, but there is a lack of a systematic summary of the knowledge status and gaps. Therefore, to address this challenge, we systematically reviewed the pharmacological mechanisms of DHM in central nervous system disorders and its potential applications in related conditions. We analyzed the therapeutic potential and current challenges of DHM to provide a reference for its development and application as a novel therapeutic agent. The review suggests that DHM possesses significant potential for the management of neurological disorders.

## 1 Introduction

Recently, the incidence of central nervous system disorders has increased, posing a substantial threat to public health. These disorders, including Alzheimer’s disease (AD), Parkinson’s disease (PD), and stroke, significantly affect the quality of life of patients. Traditional treatment methods, such as medication and surgical intervention, although able to alleviate symptoms to some extent, often have significant side effects and limited efficacy, leading to reduced patient compliance. Consequently, it is imperative to identify novel therapeutic strategies to effectively manage these intricate neurological disorders.

Dihydromyricetin (DHM), a natural product, has received increasing attention in recent years. DHM is a flavonoid compound derived from the traditional Chinese medicinal plant *Ampelopsis grossedentata*, and flavonoids yield promise in the management of neurodegenerative disorders (Minocha et al., 2022). In recent years, DHM has garnered significant attention because of its diverse pharmacological properties. Beyond its application in neurodegenerative disorders, such as AD and PD, the potential therapeutic effects of DHM in other central nervous system pathologies are increasingly being acknowledged by the research community. A growing number of preclinical and clinical studies have begun to focus on its potential applications in treating central nervous system disorders. For example, DHM has been shown to alleviate neuronal damage and improve cognitive function (Hong et al., 2025; Tao et al., 2022). In addition, the protective effects of DHM against disorders such as PD and stroke are continuously being validated, providing a theoretical basis for its clinical application in the treatment of central nervous system disorders. The molecular studies support the preclinical studies. Indeed, DHM acts via multiple mechanisms, including oxidative stress inhibition, inflammatory response regulation, and neuronal survival promotion (He et al., 2024; Wei et al., 2024).

Overall, DHM, a natural compound with multiple pharmacological effects, shows great potential for the treatment of central nervous system disorders. Yet, a summary of the available evidence for the benefits of DHM in neurological conditions is lacking. Reviewing the available evidence could help define the research gaps. This article explores the pharmacological effects of DHM, primarily in central nervous system disorders, and analyzes its application prospects to provide a reference for research and clinical practice in related fields (Table 1).

**TABLE 1:** Challenges and future directions for DHM clinical translation.

Aspect	Current status	Limitations	Future strategies
Bioavailability	Low solubility (0.2–0.32 mg/mL in water); rapid degradation at pH > 6.0	Poor BBB penetration; unstable in alkaline conditions	Nanocarriers (e.g., liposomes), prodrug design
Mechanistic Complexity	Multitarget effects (NLRP3, NF-κB, Nrf2, AMPK)	Potential off-target effects; unclear dominant pathways	Systems pharmacology approaches; CRISPR screening
Clinical Evidence	Preclinical studies only (rodent models)	Lack of human trials; unvalidated dosing regimens	Phase I/II trials for AD/PD; biomarker development
Combination Therapy	Synergistic effects with salvianolic acid B (α-syn clearance)	Drug-drug interaction risks	Screening DHM-drug combinations (e.g., metformin for insulin resistance)
Safety	No significant toxicity was reported in animals (≤100 mg/kg)	Long-term safety unknown	Toxicokinetic studies; organ-specific toxicity profiling

## 2 DHM

### 2.1 Chemical characteristics and sources of DHM

#### 2.1.1 Structural characteristics of DHM

**FIGURE 1:**
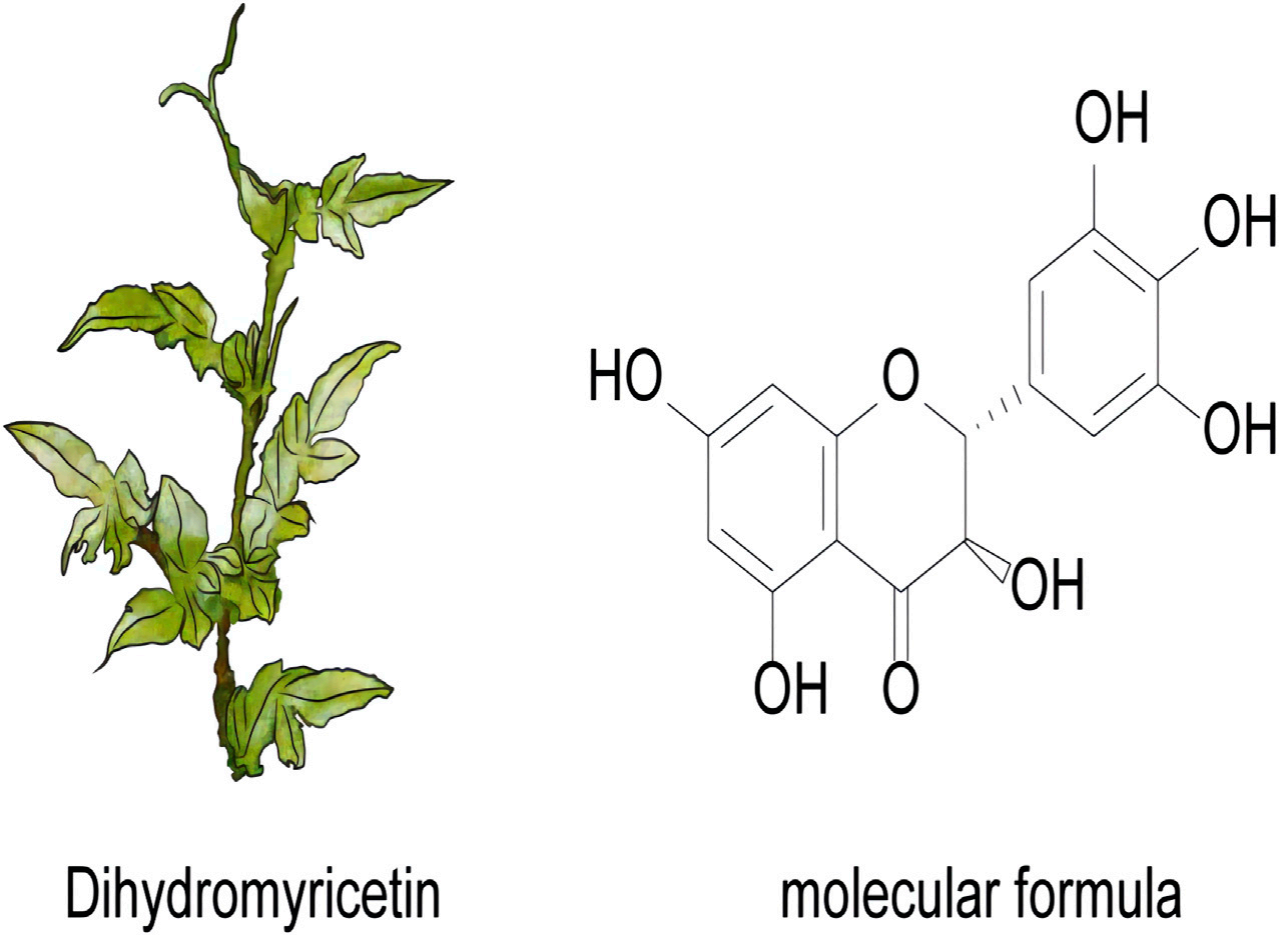
The plant and molecular formula of dihydromyricetin.

DHM is a naturally occurring flavonoid compound primarily extracted from the plant *Ampelopsis grossedentata*, commonly referred to as Tengcha. Its chemical structure belongs to the dihydroflavonoid class, and it has various biological activities. The molecular formula of DHM is C_15_H_12_O_7_, which contains multiple hydroxyl groups and one ether bond in its molecular structure, thus exhibiting excellent antioxidant and anti-inflammatory properties *in vivo* (Figure 1). DHM demonstrates a range of biological activities, including antitumor, anti-inflammatory, and antioxidant effects, through the modulation of various signaling pathways. Furthermore, the structural composition of DHM, characterized by multiple hydroxyl groups, confers relatively high solubility in aqueous environments. However, its bioavailability remains limited, particularly within the gastrointestinal tract, where degradation occurs rapidly (Sun et al., 2021a). Table 2 presents methods that were explored to improve the delivery of DHM.

**TABLE 2:** Techniques to improve the bioavailability of DHM.

Techniques	Main benefits	References
Protein nanoparticles	Improved stability, targeting, and bioavailability	Zhang et al. (2022)
Chitosan-based nanoparticles	Enhanced absorption, and biocompatibility	He et al. (2024)
PEGylated liposomes	Controlled release and increased solubility	He et al. (2024)
SEDDS/Solid SEDDS	Lymphatic transport and higher absorption	Dong et al. (2023), Wang et al. (2019)
Gastric floating dosage forms	Prolonged gastric retention, and better absorption	Molaveisi et al. (2024), Zhang et al. (2022)
Cyclodextrin complexes	Increased water solubility	He et al. (2024)
Phospholipid complexes/acylation	Enhanced membrane permeability	He et al. (2024)
Hydrogels	Sustained release and improved stability	He et al. (2024), Zhang et al. (2022)
Extracellular vesicles	High targeting and biocompatibility	He et al. (2024)
Nanocochleates/self-nanoemulsions	Improved solubility and protection from degradation	Molaveisi et al. (2024)

#### 2.1.2 Main sources and extraction methods

The main source of DHM is *Ampelopsis grossedentata*, a plant widely distributed in southern China and often used as a traditional beverage. In recent years, in-depth studies of the pharmacological effects of DHM have gradually received attention. Its biological activities include antioxidant, antibacterial, antiviral, anti-inflammatory, anticancer, and neuroprotective effects (Wang et al., 2017). Although DHM has many beneficial effects, its water solubility and stability limit its applications. There are reports indicating that the solubility of DHM in ethanol, hot water, and cold water is 170 mg/mL at 25 °C, 20 mg/mL at 80 °C, and 0.2–0.32 mg/mL at 25 °C, respectively (Wang et al., 2016). Its stability is greatly affected by pH. Under alkaline conditions, especially in the pH range of 6.0–8.0, DHM is prone to oxidation and degradation, whereas, under acidic conditions (pH range of 1.0–5.0), it is relatively stable (Hou et al., 2017), resulting in a low bioavailability of DHM (Liu et al., 2019). A range of delivery systems, such as solid dispersions, nanocapsules, microemulsions, cyclodextrin inclusion complexes, co-crystallization, phospholipid complexes, and chemical or enzymatic acylation, can effectively enhance the solubility and bioavailability of DHM (Mohapatra et al., 2021). The primary methodologies for extracting DHM include solvent- and ultrasound-assisted extractions. Using 70% ethanol for ultrasonic extraction at 70 °C can effectively improve the extraction rate of DHM by up to 21.42% (Xie et al., 2019).

During the extraction process, researchers have observed a significant correlation between the antioxidant activity of DHM and the specific methods employed. The extract was subjected to high-performance liquid chromatography analysis, enabling effective separation and identification of DHM and its related compounds. These extraction methods not only increase the yield of DHM but also lay the foundation for subsequent pharmacological research (Umair et al., 2022). In addition, the metabolic processes of DHM in the body have attracted widespread attention. It undergoes metabolic processes, such as reduction, dehydroxylation, and glucuronidation, in the body, which may affect its biological activity and efficacy (El-Haj et al., 2018).

In conclusion, DHM, a significant natural compound, holds considerable potential for application in treating central nervous system disorders, owing to its distinctive chemical structure and extensive biological activity. By optimizing the extraction method and conducting in-depth research on its pharmacological mechanisms, DHM is expected to become an important candidate for future drug development.

## 3 Pharmacological effects of DHM

**FIGURE 2:**
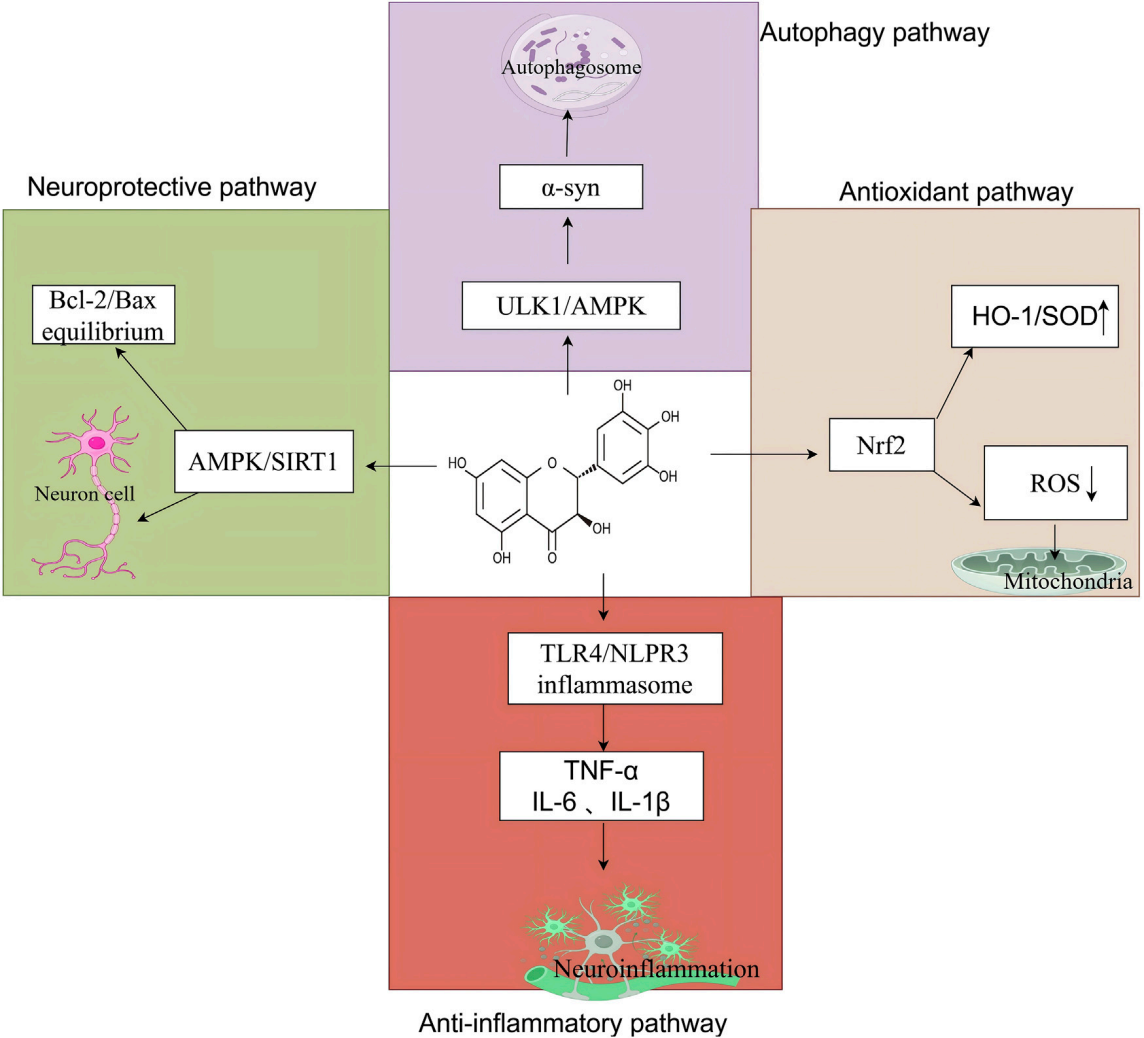
The core mechanism of DHM in neurological disorders, created using Biorender.com. The symbol “↓” indicates downregulation, and “↑” indicates. Upregulation (α-syn, α-synuclein; ULK1, Unc-51-like autophagy-activating kinase 1; AMPK, adenosine 5′-monophosphate-activated protein kinase; HO-1, heme oxygenase-1; SOD, superoxide dismutase; Nrf2, nuclear factor erythroid 2-related factor 2; ROS, reactive oxygen species; TLR4, toll-like receptor 4; NLRP3, nucleotide-binding oligomerization domain-like receptor family, pyrin domain-containing 3; TNF-α, tumor necrosis factor-alpha; IL-6, interleukin-6; IL-1β, interleukin-1 beta; Bcl-2, B-cell lymphoma 2; Bax, Bcl-2-associated X protein; SIRT1, sirtuin 1).

DHM is a naturally occurring flavonoid derived from the traditional Chinese medicinal herb *Ampelopsis grossedentata*. Recently, it has garnered significant attention from the scientific community owing to its diverse pharmacological properties. Research indicates that DHM exhibits various pharmacological effects, including antioxidant and anti-inflammatory properties, reduced apoptosis, mitigation of insulin resistance, alleviation of atherosclerosis, and neuroprotective effects (Figure 2). These findings offer a theoretical foundation for its potential applications in treating central nervous system disorders (Li et al., 2017; Martinez-Coria et al., 2019; Zhang et al., 2018) (Table 3).

**TABLE 3:** Pharmacological mechanisms of dihydromyricetin (DHM) in neurological disorders.

Mechanisms	Key targets/Pathways	Evidence *In Vitro*	Evidence *In Vivo*
Anti-inflammatory	TRL4/NF-κB suppression		Ge et al. (2019), Huang et al. (2022), Jiang et al. (2025), Pei et al. (2023), Zhang et al. (2023)
	IL-1β and other inflammatory mediator reduction	Feng et al. (2018), Jiang et al. (2025), Wu et al. (2019)	Feng et al. (2018), Ren et al. (2018), Wei et al. (2024)
	NLRP3 inflammasome inhibition	Sun et al. (2020), Wei et al. (2022)	Shi et al. (2025), Zhang and Tang (2023)
	Nrf2 inhibition	Hu et al. (2018)	Chu et al. (2018)
Antioxidant	SOD upregulation	Mu et al. (2016), Birben et al. (2012)	
	Nrf2/HO-1 activation	Jiang et al. (2014)	Liu et al. (2017), Zhang et al. (2021)
	NOX2/NOX4	Tao et al. (2022)	
	Sirt3-FOXO3a		Liu et al. (2016)
Anti-apoptotic	Increase Bcl-2 and decrease Bax/caspase-3	Zhang et al. (2021), Zhang et al. (2019)	Hou et al. (2015), Li et al. (2023), Liu et al. (2018a), Liu et al. (2018b), Mu et al. (2016), Sun et al. (2019), Tao et al. (2022), Zhang et al. (2021)
Alleviation of insulin resistance	PI3K/Akt, GLUT4 translocation	He et al. (2019), Martinez Baez et al. (2023), Mengxi et al. (2019)	Burda and Oleszek (2001), Liu et al. (2018b)
	Promotes autophagy	Shi et al. (2015)	
	PLC–CaMKK–AMPK signaling pathway		Hou et al. (2021)
Alleviation of atherosclerosis	Decreased plaque formation	Sun et al. (2021a), Wu et al. (2018), Zhang and Tang. (2023), Zhang et al. (2023)	Yang et al. (2020)
Neuroprotective effects	Reduction of infarct size and symptoms		Xie et al. (2022)
	Alleviate mitochondrial dysfunction	Wang et al. (2024)	Li et al. (2019)
	Protect dopaminergic neurons		Sugumar et al. (2019), Yans et al. (2015)

### 3.1 Anti-inflammatory effect

DHM demonstrates significant neuroprotective effects by modulating TLR4/NF-κB signaling, a central pathway driving neuroinflammation in neurological disorders. Indeed, the TLR4/NF-κB axis amplifies neuroinflammation through TLR4 activation and NF-κB nuclear translocation (Anilkumar and Wright-Jin, 2024; Hanke and Kielian, 2011; Shih et al., 2015). In AD, TLR4/NF-κB hyperactivation promotes amyloid-β toxicity and microglial inflammation (Pei et al., 2023). In stroke, early NF-κB inhibition reduces infarct size, while delayed inhibition is ineffective (Anilkumar and Wright-Jin, 2024). DHM suppresses TLR4/NF-κB signaling through direct TLR4/MyD88 inhibition, leading to decreased TLR4 and MyD88 protein expression, decreased phosphorylation of IκBα and p65, and reduced TNF-α, IL-6, IL-1β, and ROS (Ge et al., 2019; Huang et al., 2022; Jiang et al., 2025; Zhang et al., 2023). DHM also binds myeloid differentiation factor 2 (MD2), preventing TLR4 dimerization (Pei et al., 2023).

At the cellular level, DHM inhibits microglial activation, suppresses the release of inflammatory mediators, such as interleukin (IL)-1β, reduces inflammatory cell infiltration associated with microglial activation, and facilitates the transition of microglia from a pro-inflammatory state to a neuroprotective phenotype (Feng et al., 2018; Wu et al., 2019). DHM mitigates microglial activation-induced neuroinflammation by inhibiting NLRP3 inflammasome activation (Feng et al., 2018). It inhibits the release of various inflammatory mediators, including tumor necrosis factor-alpha (TNF-α) and IL-6, thereby reducing the inflammatory response (Jiang et al., 2025). DHM reduced IL-6 levels in serum and increased IL-2 levels (Feng et al., 2018). In mice rendered obese through a high-fat diet, DHM significantly decreased serum levels of IL-1β, IL-6, TNF-α, and MCP-1 (Feng et al., 2018). Furthermore, empirical studies have shown that DHM markedly suppresses the release of inflammatory mediators, including nitric oxide, prostaglandin E2, inducible nitric oxide synthase, and COX-2 (Ren et al., 2018; Wei et al., 2024). In addition, DHM alleviates neuroinflammation by inhibiting the activation of the NLRP3 inflammasome. *In vitro* experiments demonstrated that glial cells treated with DHM exhibited significantly reduced levels of inflammatory factors, indicating a potential regulatory role in neuroinflammation (Shi, et al., 2025). DHM demonstrates significant anti-inflammatory effects in hepatic inflammation models, markedly reducing inflammatory markers in the liver (Zhang et al., 2023). The activation of the inflammasome requires pathogen-associated or injury-related molecules to initiate its assembly (Yang et al., 2018a; Zhou et al., 2016). DHM can reduce the production of IL-1β and IL-18 through activation of the SIRT1 signaling pathway, thereby suppressing the NLRP3 inflammasome (Sun et al., 2020). IL-1β is considered the most important mediator in post-traumatic inflammatory response, as it reaches its peak within hours of brain tissue damage and promotes the release of other cytokines, activating nearby steady-state microglia and recruiting additional inflammatory cells (McKee and Lukens, 2016). DHM may inhibit NLRP3 activation through the TLR4/protein kinase B (Akt)/HIF1α/NLRP3 pathway (Wei et al., 2022). In a mouse model of acute kidney injury, DHM enhances antioxidant capacity by activating the Nrf2 signaling pathway, thereby inhibiting the renal inflammatory response and demonstrating its potential in treating inflammatory diseases (Zhang and Tang, 2023).

The NF-κB transcription factor is a pivotal component of the inflammatory response, modulating immune activity by inducing the expression of cytokine and chemokine genes (Wu et al., 2019). NF-κB activation is a prerequisite for NLRP3 activation (Yang et al., 2018b; Zhou et al., 2016), and DHM-mediated inhibition of NF-κB plays a central role in its anti-inflammatory mechanism. DHM attenuates inflammation by inhibiting the phosphorylation of NF-κB and subsequent nuclear translocation of p65 (Tang et al., 2016). In lipopolysaccharide-stimulated mouse glial cells, DHM inhibits neuroinflammation by downregulating the NF-κB signaling pathway and reducing the phosphorylation levels of the STAT3 nuclear translocation site and JAK2–STAT3 signaling (Weng et al., 2017). DHM also inhibits the inflammatory response and hippocampal cell apoptosis while improving cognitive function by upregulating the adenosine monophosphate-activated protein kinase (AMPK)/SIRT1 signaling pathway (Sun et al., 2019). The regulatory effect of this pathway is primarily achieved through downregulation of transcription factors NF-κB and AP-1, as well as modulation of histone acetylation (Xue et al., 2012). Additionally, SIRT1 has been shown to directly deacetylate NF-κB, thereby reducing the acetylation of p65 and suppressing its transcriptional activity and proinflammatory gene expression (Baker, Hayden and Ghosh, 2011; Matsushita et al., 2013). Cys46 has been identified as the binding site responsible for DHM-mediated inhibition of the NF-κB signaling pathway (Li et al., 2015).

Nrf2 plays a critical role in both inflammatory and oxidative stress responses. Nrf2-related signaling promotes the generation of reactive oxygen species (ROS), which serve as important inflammatory mediators (Wu et al., 2018). DHM suppresses inflammation induced by palmitic acid in human umbilical vein endothelial cells by inhibiting caspase-1 cleavage and the subsequent maturation and release of IL-1β. This mechanism is hypothesized to be associated with the Nrf2 signaling pathway (Hu et al., 2018). In a rheumatoid arthritis rat model, DHM significantly reduces inflammation by activating the Nrf2 pathway, which lowers levels of IL-1β, IL-6, TNF-α, and cyclooxygenase-2 (COX-2) (Chu et al., 2018).

### 3.2 Antioxidant effect

DHM exerts significant protective effects against atherosclerosis, tumorigenesis, apoptosis, and neurodegenerative diseases by regulating this balance (Birla, Minocha, Kumar, Misra and Singh, 2020; Chen et al., 2021a; Hu et al., 2018; Martinez-Coria et al., 2019). Experimental evidence shows that DHM regulates redox balance through multitarget action. First, DHM significantly enhances the catalytic activity of superoxide dismutase (SOD), promoting the conversion of O_2_•^-^ to H_2_O_2_ (Mu et al., 2016). In cardiac fibroblasts stimulated with angiotensin II, DHM administration markedly reduced ROS and malondialdehyde (MDA) levels, a lipid peroxidation end-product, and there is a significant enhancement in total antioxidant capacity and SOD activity (Song et al., 2017). Subsequent studies have demonstrated that DHM significantly mitigates oxidative damage caused by sodium nitroprusside in human umbilical vein endothelial cells. This protective effect is mediated through activation of the phosphatidylinositol 3-kinase (PI3K)/Akt/FoxO3a signaling pathway, as evidenced by a reduction in intracellular ROS production, decreased MDA levels, and increased SOD activity (Birben et al., 2012).

DHM specifically inhibits the expression of NADPH oxidase subtypes NOX2 and NOX4 to regulate ROS generation. In the oxygen-glucose deprivation/reoxygenation (OGD/R) model, HT22 cells treated with DHM showed not only an increase in SOD activity and a decrease in MDA content but also a significant downregulation of NOX2 and NOX4 protein expression, which is closely related to the activation of the Wnt/β-catenin signaling pathway (Tao et al., 2022). Similarly, in hippocampal neurons damaged by OGD/R, DHM inhibits NOX2/NOX4 expression through the Nrf2/HO-1 pathway while increasing SOD and reducing glutathione levels (Zhang et al., 2021). Animal experiments have shown that DHM can significantly improve the oxidative stress status of LDLr^−/−^ mice fed a high-fat diet, and its mechanism involves the inhibition of NOX2 expression and recovery of antioxidant enzyme activities, such as glutathione, SOD, and catalase (Liu et al., 2017).

At the molecular regulatory level, DHM activates the Nrf2 signaling pathway through multiple mechanisms: on the one hand, it induces the expression of autophagy adaptor protein p62, promotes the formation of the p62–Keap1–LC3II complex, accelerates Keap1 degradation, and thus relieves Nrf2 inhibition (Qiu et al., 2017); on the other hand, the activation of Akt and ERK kinases facilitates the nuclear translocation of Nrf2, thereby enhancing the expression of its downstream target gene, *H O -1* (Luo et al., 2017). In addition, DHM inhibits oxidative stress by regulating the AMPK/glucose transporter 4 (GLUT4) signaling axis, specifically by suppressing AMPK hyperphosphorylation and promoting GLUT4 membrane translocation (Jiang et al., 2014). Regarding neuroprotection, DHM significantly alleviates neuronal oxidative damage induced by low-pressure hypoxia through the Sirt3–FOXO3a pathway (Liu et al., 2016). Structure-activity relation analysis showed that the synergistic antioxidant system composed of the C-ring 3-hydroxyl and B-ring 3,4-dihydroxy groups in the DHM molecule is the structural basis for efficient free radical scavenging (Burda and Oleszek, 2001).

### 3.3 Reduction of cell apoptosis

Apoptosis involves two primary pathways: the intrinsic and extrinsic pathways. The intrinsic pathway (or mitochondrial-mediated) is activated by intracellular stressors like ROS, DNA damage, or hypoxia (Kannan and Jain, 2000; Ryter et al., 2007). The key events are the disruption of the mitochondrial membrane integrity, triggering cytochrome c release, regulation of the mitochondrial permeability through the Bcl-2 family proteins (anti-apoptotic Bcl-2 vs pro-apoptotic Bax), and the formation of the apoptosome by cytochrome c, activating caspase-9 and downstream effector caspases (e.g., caspase-3) (Kannan and Jain, 2000; Mustafa et al., 2024; Ryter et al., 2007). On the other hand, the extrinsic pathway (or death receptor-mediated) is initiated by extracellular ligands (e.g., TNF-α, FasL) binding to death receptors (e.g., TNFR, Fas). The key events include receptor oligomerization to recruit adaptor proteins (FADD/TRADD) and procaspase-8, forming the DISC, caspase-8 activation cleaves effector caspases or amplifies intrinsic signaling via Bid cleavage, and ROS enhances extrinsic pathway sensitivity by upregulating death receptors and ligand expression (Mustafa et al., 2024).

DHM counters apoptosis through multi-pathway regulation. 1) Suppression of intrinsic apoptosis through mitochondrial protection. In cerebral ischemia-reperfusion models (OGD/R-induced HT22 cells), DHM decreases the Bax/Bcl-2 ratio, caspase-3 activation, cytochrome c release, and mitochondrial permeabilization (Zhang et al., 2021; Zhang et al., 2019). DHM also upregulates Nrf2 nuclear translocation, enhancing antioxidant genes (HO-1, SOD). Brusatol (Nrf2 inhibitor) reverses DHM’s anti-apoptotic effects, confirming Nrf2 dependence (Zhang et al., 2021). 2) Modulation of extrinsic apoptosis. DHM reduces TNF-α and Fas expression in endothelial cells under oxidative stress and attenuates caspase-8 and caspase-3 cleavage in neuronal and vascular models (Zhang et al., 2021; Zhang et al., 2019). 3) Cross-pathway signaling. In HUVECs, DHM phosphorylates Akt and FoxO3a, promoting nuclear exclusion of FoxO3a (pro-apoptotic transcription factor). This effect is blocked by PI3K inhibitor LY294002, confirming pathway dependency (Zhang et al., 2021).

The antiapoptotic effect of DHM is primarily manifested through its influence on the activation of apoptosis-related signaling pathways and modulation of apoptosis-associated protein expression. The Bcl-2 protein family is integral to the mitochondrial pathway of cellular apoptosis, with the proapoptotic protein Bax and antiapoptotic protein Bcl-2 serving as crucial constituents of this family. Equilibrium between these two proteins is essential for maintaining normal apoptotic processes under physiological conditions (Youle and Strasser, 2008). Furthermore, caspase-3, a protein responsible for cellular apoptosis, serves as an indicator of the extent of apoptosis through quantitative variation (Wu et al., 2017). DHM markedly decreases the prevalence of apoptotic cells across various injury models and exerts its effects by downregulating proapoptotic proteins, such as Bax and caspase-3, while upregulating the antiapoptotic protein Bcl-2 (Hou et al., 2015; Li et al., 2023a; Liu et al., 2018a; Liu et al., 2018b; Mu et al., 2016; Sun et al., 2019; Tao et al., 2022; Zhang et al., 2021). Sun et al. suggested that the antiapoptotic effect of DHM is achieved by upregulating the AMPK/SIRT1 pathway (Sun et al., 2019), whereas Jiang et al. suggested that DHM inhibits cell apoptosis through the AMPK/GLUT4 signaling pathway (Jiang et al., 2014). In addition, Hu et al. found that knocking out the *Nrf2* gene counteracted DHM’s protective effect on palmitic acid-induced cell apoptosis, suggesting that the antiapoptotic effect of DHM may be related to Nrf2-related pathways (Hu et al., 2018). DHM significantly inhibits apoptosis by activating the AMPK/SIRT1 pathway (Sun et al., 2019). In addition, DHM exerts antiapoptotic effects through the AMPK/GLUT4 signaling pathway (Jiang et al., 2014). Notably, the Nrf2 signaling pathway has also been confirmed to be involved in the antiapoptotic mechanism of DHM. Knocking out the *Nrf2* gene can significantly weaken the protective effect of DHM against palmitic acid-induced cell apoptosis, suggesting a key role for the Nrf2 pathway in the antiapoptotic function of DHM (Hu et al., 2018).

In an ischemia-reperfusion model, the application of DHM significantly reduced the number of apoptotic cells, improved cell survival rates, and demonstrated its potential for neuroprotection (Wang et al., 2024). In an ischemia-reperfusion injury model, DHM has been shown to substantially decrease the prevalence of apoptotic cells and enhance cell survival rates, indicating its potential efficacy in neuroprotection (Wei et al., 2019). This effect may be related to DHM’s regulation of Bcl-2 family proteins, the inhibition of ROS generation, and the activation of related signaling pathways.

### 3.4 Alleviation of insulin resistance

Insulin resistance is a central pathological characteristic of type 2 diabetes and metabolic syndrome, and its manifestation is intricately linked to dysregulation of the insulin signaling pathway. In recent years, DHM has attracted considerable attention because of its significant role in improving insulin resistance. DMY exhibits potential therapeutic value by regulating the insulin signaling pathway, promoting glucose uptake, inhibiting inflammatory responses, and enhancing antioxidant capacity through various mechanisms.

#### 3.4.1 Regulatory effect of DHM on insulin signaling pathway

DHM markedly increases the phosphorylation of insulin receptor substrates (IRS)-1 and Akt, thereby augmenting the biological activity of insulin and enhancing glucose metabolism (Martinez Baez et al., 2023). Specifically, DHM upregulates the expression of IRS, thereby enhancing insulin signaling. For instance, the phosphorylation of IRS is pivotal in insulin signaling, influencing the translocation of GLUT4 and facilitating glucose uptake (He et al., 2024). DHM improves insulin resistance by activating the PI3K/Akt pathway and promoting glucose uptake and metabolism. Under high-glucose and insulin conditions, DHM significantly increases glucose consumption and intracellular glycogen synthesis, indicating its potential therapeutic effect in improving insulin resistance (Mengxi et al., 2019).

IRS-1 (Y612) is thought to enhance the intracellular transmission of insulin signals. DHM upregulates tyrosine phosphorylation of IRS-1 (Y612), thereby improving insulin resistance (He et al., 2019).

#### 3.4.2 Activation of AMPK signaling pathway by DHM

DMY promotes Akt phosphorylation and activates the AMPK signaling pathway, thereby enhancing GLUT1-mediated glucose transport and maintaining glucose homeostasis (Le et al., 2016). In addition, DHM promotes autophagy in skeletal muscle cells and increases insulin sensitivity by activating the AMPK–PGC-1α–Sirt3 signaling pathway (Shi et al., 2015). In a dexamethasone-induced obesity-related insulin resistance model, DHM significantly increases adipocyte insulin sensitivity by inhibiting ERK-mediated phosphorylation of the PPARγ serine 273 site, promotes glucose uptake, and reduces lipid accumulation (Liu et al., 2018b).

DHM also promotes the phosphorylation of Akt, a key AMPK substrate. AMPK, a sensitive glucose sensor, facilitates glucose transport via GLUT1 to maintain glucose homeostasis. Furthermore, DHM inhibits glycogen synthase kinase-3β (GSK-3β), thereby delaying the progression of insulin resistance (Burda and Oleszek, 2001).

#### 3.4.3 Inhibitory effect of DHM on inflammatory response

DHM alleviates insulin resistance by inhibiting inflammatory responses, demonstrating its potential application in treating metabolic diseases (Pei et al., 2022). Other natural compounds, such as flavonoids, exert similar effects. Flavonoids alleviate insulin resistance and improve insulin signaling by inhibiting inflammatory signaling pathways (Che et al., 2019). These compounds enhance insulin signaling efficiency by regulating the phosphorylation status of IRS (Pei et al., 2022).

Hou et al. demonstrated *in vivo* and *in vitro* that DHM counteracts inflammation-induced insulin resistance via the PLC–CaMKK–AMPK signaling pathway and identified PLC as a potential DHM target in the mitigation of insulin resistance (Hou et al., 2021).

#### 3.4.4 Potential application of DHM in treating insulin resistance

Zhou et al. reported that DHM alleviates insulin resistance by inhibiting the expression of FLCN and FNIP1, thereby preventing the reduction in slow-twitch muscle fibers (Zhou et al., 2017). Shi et al. found that DHM enhances skeletal muscle cell sensitivity to insulin by promoting autophagy, an effect possibly mediated by the AMPK-PGC-1α-Sirt3 signaling pathway (Shi et al., 2015).

Ning proposed that the mechanism by which DHM reduces insulin resistance may involve the enhancement of antioxidant activity, which alleviates alloxan-induced damage to the liver and pancreatic beta cells, a process closely associated with hepatic glycogen synthesis and insulin production (Le et al., 2016).

In summary, DHM and other natural compounds exhibit substantial potential for improving insulin resistance by targeting key regulatory nodes within the insulin signaling pathway and enhancing overall insulin sensitivity.

### 3.5 Alleviation of atherosclerosis

DHM significantly inhibits endothelial cell damage induced by ROS and oxidized low-density lipoproteins (oxLDL). Hu et al. demonstrated that, in a palmitic acid-induced human umbilical vein endothelial cell injury model, DHM effectively suppresses inflammation, reduces oxidative stress, and decreases cell apoptosis by activating the Nrf2 signaling pathway, thereby exerting endothelial protective effects (Zhang and Tang, 2023; Zhang et al., 2023). In addition, Yang et al. found that DHM significantly inhibits atherosclerotic plaque formation in apolipoprotein E-deficient mice via suppression of miR-21 expression, which regulates the DDAH1–ADMA–eNOS–NO signaling pathway, promotes nitric oxide production, and improves lipid metabolism (Yang et al., 2020). In a TNF-α-induced endothelial dysfunction model, DHM also reverses dysregulation of the DDAH1/ADMA/NO signaling pathway and improves endothelial function by inhibiting miR-21 expression (Yang et al., 2018a).

DHM significantly modulates inflammatory responses. Experimental data indicate that DHM reduces macrophage and CD4^+^ T-cell infiltration in the vascular wall of apolipoprotein E-deficient mice while downregulating proinflammatory gene expression (Yang et al., 2020). At the molecular level, DHM improves mitochondrial function, reduces oxidative stress, and inhibits NLRP3 inflammasome activation in oxLDL-stimulated macrophages, thereby suppressing the release of inflammatory cytokines (Sun et al., 2021a).

DHM exerts multiple protective effects against oxidative stress. DHM not only directly neutralizes ROS and enhances the activity of antioxidant enzymes, such as SOD but also augments cellular antioxidant defense mechanisms by activating the Nrf2/HO-1 signaling pathway (Wu et al., 2018; Zhang and Tang, 2023; Zhang et al., 2023). In macrophages exposed to ox-LDL, DHM significantly inhibits NLRP3 inflammasome activation and mitigates inflammation-mediated endothelial injury by upregulating SIRT3 expression (Sun et al., 2021a).

DHM plays a protective role in lipid metabolism by inhibiting abnormal cholesterol accumulation and foam cell formation. Experimental evidence indicates that DHM significantly reduces oxLDL-induced lipid deposition in macrophages, enhances mitochondrial function, and simultaneously alleviates oxidative stress and inflammation (Sun et al., 2021b). These combined effects inhibit the formation and progression of atherosclerotic plaques.

Several studies have highlighted the synergistic effects of DHM with other natural compounds. For instance, hydroxytyrosol and pomegranate components delay the progression of atherosclerosis by improving endothelial function and regulating lipid metabolism (Guo et al., 2020; Quiros-Fernandez et al., 2019), whereas α-lipoic acid protects vascular endothelium through potent antioxidant and anti-inflammatory actions (Cuerda et al., 2011). A compound preparation of red koji rice and polymethoxyflavones has also been shown to reduce atherosclerosis risk by improving lipid mass spectrometry profiles and endothelial function (Cimaglia et al., 2019).

### 3.6 Neuroprotective effect

#### 3.6.1 Neuroprotective effect of DHM in ischemic brain injury

DHM improves neurological symptoms and reduces cerebral infarction volume. These effects are primarily attributable to the inhibition of ROS and inflammatory cytokine release, thereby attenuating neuronal apoptosis resulting from ischemia-reperfusion injury (Xie et al., 2022). Animal studies have demonstrated that DHM treatment enhances neuronal survival while downregulating apoptotic markers (Xie et al., 2022).

DHM acts via a dual regulatory mechanism: it inhibits oxidative stress and neuroinflammation, thereby reducing neuronal apoptosis, and simultaneously promotes neural repair by enhancing the expression of nerve growth factor and brain-derived neurotrophic factor (BDNF) (Uddin et al., 2020; Zhan et al., 2023). The bidirectional nature of this mechanism highlights its therapeutic promise in neurodegenerative disorders.

At the molecular level, DHM mitigates oxidative stress-induced mitochondrial dysfunction by activating the SIRT1/FOXO3a signaling pathway (Wang et al., 2024). This effect was corroborated in a myocardial ischemia-reperfusion injury model, where DHM improved mitochondrial integrity via SIRT3 upregulation, reduced oxidative damage, decreased infarct size, and enhanced cardiac function (Li et al., 2019). These mitochondrial benefits were also confirmed *in vitro* (Li et al., 2019) (Table 4).

**TABLE 4:** Molecular targets of DHM in neuroprotection.

Target	Biological role	DHM’s action *In Vivo*	Disease relevance	References
NLRP3	Inflammasome activation	Inhibits assembly; ↓ IL-1β/IL-18 via SIRT1	AD, PD, stroke	Feng et al. (2018), Zhou et al. (2016)
SIRT1/AMPK	Energy metabolism, autophagy	↑ Autophagy flux; ↓ mTOR signaling	AD, insulin resistance	Jiang et al. (2014), Sun et al. (2019)
Nrf2/HO-1	Antioxidant response	↑ SOD/GSH; ↓ ROS-mediated damage	Stroke, diabetic neuropathy	Hu et al. (2018), Zhang et al. (2021)
BDNF/TrkB	Synaptic plasticity	Restores GABAergic transmission; ↑ neuron survival	Depression, anxiety	Ge et al. (2019), Liang et al. (2014)
α-Synuclein	Protein aggregation	Binds oligomers; promotes fibril depolymerization	PD	Ardah et al. (2020), Wu et al. (2019)

In PD models, DHM demonstrated specific neuroprotective effects on dopaminergic neurons. It significantly improves dopaminergic neuron survival by reducing oxidative stress and neuroinflammation while promoting mitochondrial biogenesis (Sugumar et al., 2019; Yans et al., 2015). In a neuronal injury model induced by OGD/R, DHM significantly attenuated oxidative damage and neuronal apoptosis in hippocampal cells by activating the Nrf2/HO-1 signaling pathway (Zhi et al., 2019).

### 3.7 Other pharmacological effects

Traditional antiplatelet and anticoagulant drugs are frequently associated with an increased risk of bleeding. However, studies have demonstrated that DHM has potential as a novel antithrombotic agent. In a FeCl_3_-induced carotid artery injury model, DHM exhibited multiple antiplatelet effects by significantly inhibiting platelet aggregation, secretion, adhesion, and spreading, as well as blocking integrin activation. In addition, DHM inhibited exocytosis, phosphatidylserine exposure, and tissue factor expression in activated endothelial cells. Mechanistic studies revealed that DHM exerts antithrombotic effects by reducing thrombin-induced calcium mobilization in platelets and endothelial cells and by inhibiting the ERK1/2 pathway and p38 phosphorylation (Chen et al., 2021b). These findings provide important evidence supporting the potential therapeutic application of DHM in cerebral infarction and other thrombotic conditions.

Regarding neurocognitive function, DHM has shown notable effects on memory enhancement. Experimental studies have demonstrated that DHM effectively alleviates memory dysfunction caused by sleep deprivation. The mechanism involves 1) significantly reducing MDA levels and increasing SOD activity and 2) promoting the expression of postsynaptic density protein 95 and BDNF (Li et al., 2019). Further studies have shown that DHM reduces oxidative stress by activating SIRT3-mediated deacetylation of FOXO3, promotes mitochondrial biogenesis, improves mitochondrial morphology and function, inhibits ROS production, and reduces hippocampal lipid peroxidation, thereby ameliorating hypoxia-induced memory impairment (Liu et al., 2017). These results suggest novel therapeutic strategies for cognitive disorders involving DHM.

## 4 Therapeutic applications in neurological disorders

DHM demonstrates multifaceted therapeutic potential across major neurological disorders through its pleiotropic mechanisms, as described above. In AD, DHM reduces Aβ plaques and tau hyperphosphorylation while enhancing cholinergic function via AMPK/SIRT1 activation. For OD, it inhibits α-syn aggregation and protects dopaminergic neurons by modulating NLRP3 inflammasome and autophagy pathways. In ischemic stroke, DHM mitigates oxidative damage and promotes neurogenesis through Wnt/β-catenin and Nrf2/HO-1 signaling. Additionally, DHM alleviates depression/anxiety and alcohol-related disorders by restoring GABAergic transmission and BDNF/TrkB signaling and improves neuropathic pain via microglial polarization (Li et al., 2017). These disease-specific effects are supported by robust preclinical evidence, as systematically summarized in Table 5.

**TABLE 5:** Therapeutic applications of DHM in neurological disorders.

Disease	Models	Key findings *In Vivo*	Proposed mechanisms	References
Alzheimer’s Disease (AD)	Aβ42-induced AD mice	↓ Aβ plaques; ↑ cognitive function via AMPK/SIRT1	Aβ clearance, anti-inflammation	Kou et al. (2016), Sun et al. (2019)
Parkinson’s Disease (PD)	MPTP-induced PD mice	↓ α-syn aggregation; protects dopaminergic neurons	Autophagy activation, NLRP3 inhibition	Ardah et al. (2020), Zhang et al. (2023)
Ischemic Stroke	MCAO rat model	↓ infarct volume by 30%–40%; improves motor function	Wnt/β-catenin, Nrf2 activation	Ding et al. (2023), Tao et al. (2022)
Depression/Anxiety	LPS-induced depression mice	↓ TNF-α/IL-6; ↑ BDNF/TrkB signaling	TLR4/NF-κB inhibition	Huang et al. (2022), Wei et al. (2022)
Neuropathic Pain	Diabetic neuropathy rats	↑ mechanical withdrawal threshold; ↓ P2X7-mediated inflammation	ALDH2 activation, M2 microglial polarization	Guan et al. (2019), Zhang et al. (2020a)

### 4.1 Application of DHM in AD

**FIGURE 3:**
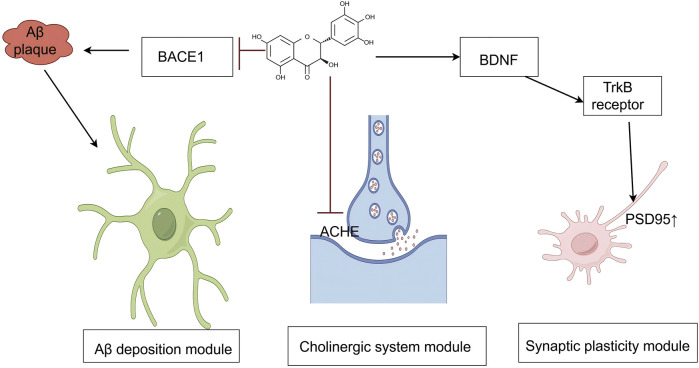
An illustration of the signaling pathways by which mechanisms of DHM in Alzheimer’s disease. The symbol “
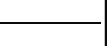
” represents inhibition, and “
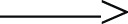
” represents promotion. (Aβ, amyloid-beta; BACE1, beta-site amyloid precursor protein cleaving enzyme 1; AChE, acetylcholinesterase; BDNF, brain-derived neurotrophic factor; TrkB, tropomyosin receptor kinase B; PSD95, postsynaptic density protein 95).

AD is among the most common neurodegenerative diseases and is characterized by abnormal deposition of β-amyloid protein (Aβ), neurofibrillary tangles, neuroinflammation, and oxidative stress. Although several pharmacological agents are currently employed to manage AD, their clinical efficacy remains limited, and adverse effects are common. Consequently, the development of new treatment strategies has become a major focus in AD research. DHM, a natural flavonoid compound, exhibits antioxidant, anti-inflammatory, and neuroprotective properties and has shown therapeutic promise in AD research (Figure 3).

#### 4.1.1 Mechanism by which DHM reduces Aβ deposition

DHM reduces Aβ deposition via a multitarget mechanism. In the production phase, DHM inhibits β-secretase 1 activity and upregulates α-secretase expression, promoting the non-amyloidogenic hydrolysis of amyloid precursor protein, thereby increasing the production of sAPPα and C83 fragments and reducing Aβ generation (Goedert and Spillantini, 2006; Jia et al., 2019b; Jia et al., 2019a; Kou et al., 2016; Naushad et al., 2019). During the aggregation phase, DHM has been shown to inhibit Aβ_40_ fibril formation and depolymerize preformed Aβ fibrils, significantly reducing their cytotoxicity in PC12 cells (Goedert and Spillantini, 2006; Jia et al., 2019b; Jia et al., 2019a; Jiang et al., 2014; Kou et al., 2016; Liang et al., 2014; Naushad et al., 2019; Shimmyo et al., 2008; Wang et al., 2016). In the clearance phase, DHM regulates intracellular redox status by activating SIRT1, upregulates neprilysin expression to promote Aβ degradation, and inhibits microglial activation and NLRP3 inflammasome expression, thereby reducing neuroinflammation and Aβ accumulation via multiple pathways (Hersh and Rodgers, 2008; Wang et al., 2016; Yans et al., 2015). In animal models of AD induced by D-galactose, DHM has demonstrated significant neuroprotective effects. Kou et al. reported that DHM effectively inhibits D-galactose-induced brain aging and neuronal apoptosis, with its mechanism closely associated with the regulation of the SIRT1-mTOR signaling pathway (Peng, Ramatchandirin, Pearah and He, 2023). Further studies have confirmed that DHM not only significantly improves cognitive impairment in model animals but also reduces brain levels of Aβ (Li et al., 2019; Liang et al., 2014). These findings suggest that DHM plays a comprehensive role in modulating the entire Aβ production-aggregation-clearance pathway and may be a key modulator of AD pathology.

#### 4.1.2 Effect of DHM on cognitive function improvement

Substantial progress has been made in understanding how DHM improves cognitive function through multiple mechanisms. Considering its antioxidant and anti-inflammatory effects, several studies have confirmed that DHM significantly reduces the levels of proinflammatory cytokines, such as TNF–α and IL-6, thereby effectively inhibiting neuroinflammation. Notably, in an Aβ_1–42_-induced AD mouse model, DHM improved learning ability, reduced neuronal apoptosis, and modulated the expression of apoptosis-related proteins, such as Bax and Bcl-2, via the AMPK/SIRT1 signaling pathway (Wang et al., 2016). In addition, its inhibition of NLRP3 inflammasome activation reduced caspase-1 production and mature IL-1β levels, further supporting its anti-inflammatory mechanism (Kou et al., 2016).

DHM also improves cholinergic neurotransmission by inhibiting acetylcholinesterase (AChE) activity. In a D-galactose-induced AD model, DHM increased the activity of antioxidant enzymes, such as catalase and SOD, reduced MDA levels, and specifically inhibited AChE, thereby reducing damage to the cholinergic system. *In vitro* studies further confirmed DHM as a non-competitive AChE inhibitor, providing mechanistic insight into its efficacy in ameliorating cognitive deficits (Hersh and Rodgers, 2008; Li et al., 2025; Sun et al., 2022).

Studies on neurotransmitters and synaptic plasticity have shown that DHM improves memory function in AD model animals. It reduces brain levels of Aβ_1–40_ and Aβ_1–42_, restores GABAergic neurotransmission, and modulates the expression of postsynaptic proteins, such as bridging integrator proteins (Liang et al., 2014). Behavioral experiments have validated the therapeutic effect of DHM in D-galactose-induced cognitive impairment models, with significant improvements observed in novel object recognition and Y-maze performance (Li et al., 2025; Wang et al., 2016), supporting its potential for clinical application in cognitive impairment associated with AD.

#### 4.1.3 Potential therapeutic strategies of DHM

In AD models induced by Aβ_1–42_, DHM has also demonstrated significant therapeutic effects. Research by Sun et al. showed that DHM improves learning ability, reduces neuronal apoptosis, and lowers levels of inflammatory cytokines, such as IL-1β, IL-6, and TNF–α (Wang et al., 2016). Mechanistic studies further revealed that DHM regulates the Bax/Bcl-2 expression balance via the AMPK/SIRT1 signaling pathway, effectively protecting neurons from Aβ-induced cytotoxicity (Yans et al., 2015). Based on the multiple mechanisms of action of DHM in AD, future treatment strategies could focus on its development as a dietary supplement or pharmaceutical agent. First, the eutectic form of DHM may enhance its water solubility and bioavailability, thereby increasing its efficacy in the central nervous system (Molaveisi et., 2024). Second, the co-administration of other natural products or drugs may potentiate the therapeutic effects of DHM, for instance, in combination with other antioxidants or anti-inflammatory agents, to achieve synergistic outcomes. In addition, optimizing the route of DHM administration, particularly through the development of nanocarrier systems capable of effectively crossing the blood-brain barrier, represents a promising direction for enhancing its clinical potential (Song et al., 2021). Through these strategies, DHM is expected to become a novel therapeutic option for the treatment of AD, offering new hope for improving patients’ quality of life.

### 4.2 Application of DHM in PD

**FIGURE 4:**
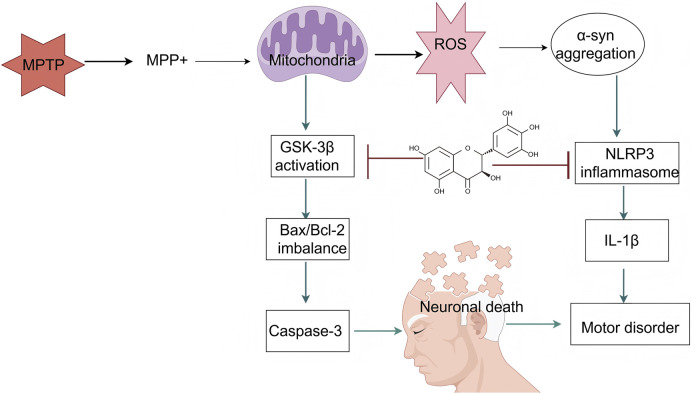
An illustration of the signaling pathways by which mechanisms of DHM in Parkinson’s disease. The symbol “
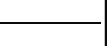
” represents inhibition, and “
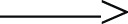
” represents promotion (MPTP, 1-methyl-4-phenyl-1,2,3,6-tetrahydropyridine; MPP+, 1-methyl-4-phenylpyridinium; ROS, reactive oxygen species; α-syn, alpha-synuclein; GSK-3β, glycogen synthase kinase-3 beta; NLRP3, NOD-like receptor protein 3; Bax, BCL2-associated X protein; Bcl-2, B-cell lymphoma 2; IL-1β, interleukin-1 beta; Caspase-3, cysteine-aspartic acid protease 3).

PD is a prevalent neurodegenerative disorder characterized by the progressive loss of dopaminergic neurons in the substantia nigra and abnormal aggregation of alpha-synuclein (α-syn). In recent years, DHM, a natural flavonoid, has demonstrated significant neuroprotective effects in PD-related research. This article systematically summarizes the applications and underlying mechanisms of DHM in PD (Figure 4).

#### 4.2.1 Regulation of dopaminergic neurons

DHM has shown considerable potential in PD research, particularly in animal models. Studies have demonstrated that DHM improves motor function in a mouse model of PD induced by 1-methyl-4-phenyl-1,2,3,6-tetrahydropyridine (MPTP). Specifically, the administration of DHM significantly increased the survival rate of dopaminergic neurons and reduced the extent of α-syn aggregation, which is closely associated with the pathophysiology of PD (Zhang et al., 2023). In one study, DHM not only significantly improved MPTP-induced motor deficits but also inhibited the expression of neuroinflammatory markers, such as IL-1β and TNF-α, in mice (Guo et al., 2020). The various biological activities of DHM have been extensively explored in preclinical studies. Research indicates that DHM exhibits antioxidant, anti-inflammatory, and neuroprotective properties, all of which are highly relevant to the treatment of PD. DHM alleviates PD-like pathology by activating the AMPK and ULK1 pathways. Specifically, DHM administration enhances AMPK activity, thereby promoting autophagy, a critical process for clearing aggregated α-syn (M. Zhang and Tang, 2023). In addition, DHM has been shown to inhibit both the aggregation and toxicity of α-syn, providing a theoretical basis for its potential as a therapeutic agent (Ardah et al., 2020). In another study, DHM reduced α-syn neurotoxicity by facilitating its transformation into a nontoxic fibrotic form, thereby offering a novel therapeutic approach for PD (Li et al., 2017).

The mechanisms underlying DHM’s therapeutic effects in PD involve multiple biological pathways. First, DHM directly interferes with disease pathology by inhibiting the aggregation and cytotoxicity of α-syn. DHM can bind to α-syn oligomers, inhibit further aggregation, and promote the depolymerization of pre-formed fibrils, thereby reducing cytotoxic effects (Ardah et al., 2020; Guo et al., 2020). Second, DHM confers neuroprotection by modulating neuroinflammatory responses. Its anti-inflammatory effects are primarily mediated through the inhibition of the NF-κB signaling pathway, which plays a pivotal role in PD-associated inflammation (Li et al., 2023b; Wei et al., 2024). In addition, the activation of autophagy by DHM constitutes another important neuroprotective mechanism. By enhancing autophagic processes, DHM facilitates the degradation of α-syn, thereby mitigating neuronal damage (Li et al., 2023a; Zhang and Tang, 2023; Zhang et al., 2023).

DHM has significant antioxidant effects and can effectively eliminate ROS in the body, thereby reducing neuronal damage caused by oxidative stress. Oxidative stress is an important factor that leads to the death of dopaminergic neurons. DHM can enhance the expression of intracellular antioxidant enzymes by activating the Nrf2 signaling pathway, thereby improving the antioxidant capacity of neurons (Nguyen et al., 2009). In addition, in MPTP-induced PD animal models, DHM reduces the production of the toxic metabolite MPP^+^ by inhibiting the activity of GSK-3β, thereby reducing ROS generation and protecting dopaminergic neurons from damage (Blandini and Armentero, 2012; Ren et al., 2018). Moreover, DHM inhibited neuroinflammatory responses. In PD, excessive microglial activation leads to the release of inflammatory factors, thereby exacerbating neuronal damage. DHM reduces the production of inflammatory factors, such as TNF-α and IL-6, by inhibiting the NF-κB signaling pathway, thereby alleviating neuroinflammation (Wei et al., 2024). The study also found that DHM reduced the number of microglia in the brains of PD mice and inhibited astrocyte-mediated neuroinflammation, further protecting dopaminergic neurons (L. Wei et al., 2024). In addition, DHM enhances the chaperone-mediated autophagy pathway, promotes the degradation of α-syn, and reduces abnormal aggregation (Goedert and Spillantini, 2006). DHM can also directly inhibit the formation of α-syn fibers and disrupt the stability of the formed α-syn fibers, protecting cells from their toxic effects (Jia et al., 2019b). Finally, DHM regulates cellular energy metabolism and enhances the survival ability of dopaminergic neurons by activating the AMPK and Akt/GSK-3β signaling pathways. DHM can increase ATP levels, reduce MPP^+^-induced cellular energy metabolism disorders, and protect neurons from damage (Guo et al., 2020; Ren et al., 2016).

#### 4.2.2 Regulation of neurotransmitters such as GABA and glutamate

In addition to protecting dopaminergic neurons, DHM has also shown positive effects in improving motor symptoms in patients with PD. Motor symptoms, including tremors, muscle stiffness, and bradykinesia, are among the most prominent manifestations of PD. Studies have shown that DHM can improve motor symptoms through multiple mechanisms. First, DHM improves motor function by promoting dopamine synthesis and release. Patients with PD have significantly reduced dopamine levels, which leads to decreased motor ability. DHM can promote the activity of key enzymes, such as tyrosine hydroxylase, in the dopamine synthesis pathway, thereby increasing dopamine synthesis and improving motor symptoms (L. Jiang et al., 2025). In addition, DHM can inhibit the activity of catechol-O-methyltransferase, increase the bioavailability of levodopa, and alleviate motor symptoms of PD (M. Wu et al., 2015). Second, DHM has shown significant improvement in motor ability in animal models. DHM can improve balance and coordination abilities in animal models of PD and reduce bradykinesia and muscle stiffness (Li et al., 2023a). These effects may be related to its antioxidant and anti-inflammatory properties, which reduce damage to the nervous system and improve motor function.

In addition, DHM improves motor control by regulating the balance of neurotransmitters, such as GABA and glutamate. DHM can reduce the excessive release of GABA and regulate the excitotoxicity of glutamate, thereby restoring normal motor functions (Guo et al., 2020). This regulatory effect may be achieved by altering the release of neurotransmitters and receptor sensitivity.

In summary, DHM has shown multifaceted potential for the treatment of PD. It significantly protects dopaminergic neurons and improves motor symptoms through various mechanisms, such as antioxidant and anti-inflammatory effects, regulation of autophagy, and enhancement of dopamine synthesis. Although the current research results are encouraging, the specific mechanism of action of DHM in PD treatment requires further exploration. Future clinical studies will help validate its efficacy and safety and provide new treatment options for patients with PD.

### 4.3 Application of DHM in stroke disease

#### 4.3.1 Post-stroke neural repair

##### 4.3.1.1 Multitarget mechanism of neuroprotective effect

Neuronal damage following ischemic stroke involves a complex pathophysiological process. Research has shown that DHM exerts neuroprotective effects through multiple pathways: 1) antioxidant effects: effectively clearing free radicals and reducing oxidative stress damage; 2) antiapoptotic effect: significantly downregulated the expression of apoptotic proteins, such as Bax and cleaved caspase-3 (Ding et al., 2023); 3) promoting regeneration function: by activating the Wnt/β-catenin signaling pathway, it promotes neuronal survival and regeneration, which has been validated in the OGD/R model (Tao et al., 2022); 4) anti-inflammatory regulation: inhibits NLRP3 inflammasome activation and reduces the release of proinflammatory factors, such as IL-1β and TNF-α (Ding et al., 2023). Notably, the latest research has found that DHM can also alleviate cell damage by regulating the SNHG10/miR-665/RASSF5 axis (Tao et al., 2022) and alleviate iron death by inhibiting the SPHK1/mTOR pathway (Zhang et al., 2023), demonstrating its multitarget therapeutic advantages.

##### 4.3.1.2 Efficacy validation in animal models

In the middle cerebral artery occlusion model, DHM exhibits significant neuroprotective effects: 1) structural protection: the treated group reduces the infarct volume by approximately 30%–40% compared with that of the control group (Li et al., 2023a); 2) functional improvement: neurobehavioral scores increased by 20%–25%, and motor coordination ability significantly improved (Li et al., 2023b); 3) time-effect relation: early administration (10–15 min after reperfusion) yields the best therapeutic effect, whereas delayed administration reduces efficacy by more than 50% (Wasan et al., 2022); 4) dose-effect relation: a dose of 100 mg/kg is significantly better than 50 mg/kg in improving neurological function and reducing infarct size (Wasan et al., 2022). These results provide important preclinical evidence for the clinical applications of DHM.

##### 4.3.1.3 Challenges and prospects faced by clinical translation

Despite the good results of animal experiments, key issues remain in the clinical translation of DHM: (1) Limitations of the model, most existing studies use young, healthy animals, failing to simulate the common complications (including diabetes and hypertension) and aging factors of clinical patients (McCann and Lawrence, 2020; Taha et al., 2022); (2) Mechanistic complexity, DHM involves multiple signaling pathways, including Wnt/β-catenin, NF-κB, and mTOR (Tao et al., 2022; Xie et al., 2022), and its synergistic mechanism requires further elucidation; (3) Lack of clinical data, only a few small-scale clinical trials have been reported, and their safety and efficacy require validation through large-scale randomized controlled trials (Åsberg et al., 2023). Future research should focus on (1) establishing animal models that more closely mimic clinical conditions, (2) systematically evaluating long-term toxicity and pharmacokinetic characteristics, and (3) determining the optimal dosing regimen, including time window, dosage, and combination therapy strategy.

DHM, a natural compound with multitarget properties, has demonstrated unique advantages in ischemic stroke treatment. It significantly improves neurological function prognosis through multiple mechanisms, including antioxidant, anti-inflammatory, antiapoptotic, and cell death pathway regulation. Although clinical translation currently faces challenges, DHM is expected to become a new option for comprehensive stroke treatment by optimizing experimental design, advancing mechanistic research, and conducting standardized clinical trials. Future studies should prioritize resolving key translational issues from laboratory to clinical settings to provide a scientific basis for developing more effective stroke treatment strategies.

#### 4.3.2 Role of cerebral hemorrhage and subarachnoid hemorrhage

Cerebral and subarachnoid hemorrhages are the two main types of stroke, often accompanied by severe neurological dysfunction. DHM has shown potential therapeutic effects in both conditions. Studies indicate that DHM alleviates neurological damage after cerebral hemorrhage by inhibiting oxidative stress and inflammatory responses. Specifically, DHM significantly reduces ROS levels in brain tissue following cerebral hemorrhage and increases endogenous antioxidant enzyme activity, thereby protecting nerve cells from damage (Li et al., 2023a).

In the subarachnoid hemorrhage model, DHM has also demonstrated neuroprotective effects. DHM enhances the expression of antioxidant enzymes, such as peroxiredoxin 2, by activating the Nrf2 signaling pathway, thereby reducing oxidative damage to nerve cells (Li et al., 2023b). In addition, DHM inhibits apoptosis and improves neurological function, supporting its potential application in subarachnoid hemorrhage treatment.

In summary, DHM holds broad potential for stroke treatment. It exerts therapeutic effects through multiple mechanisms, including antioxidant, anti-inflammatory, and neurorepair activities. This study provides new insights and directions for future clinical stroke treatment, warranting further research and clinical validation.

### 4.4 Role of DHM in mental illness

DHM, a natural flavonoid, has shown promising applications in mental disorder research. Studies suggest that DHM has significant therapeutic potential in mental illnesses such as anxiety and depression. Anxiety disorder is one of the most prevalent psychological disorders in the United States, with many patients exhibiting drug resistance or severe side effects from existing treatments. Identifying new therapeutic strategies is, therefore, critical.

#### 4.4.1 Role of DHM in anxiety disorder treatment

DHM significantly improves anxiety-related behavior by regulating GABAergic neurotransmission and inhibiting neuroinflammation. DHM restores GABAergic neurotransmission in mice, increases intracellular ATP levels, and enhances gephyrin expression, thereby improving anxiety-like behaviors (Silva et al., 2020). Gephyrin functions as a postsynaptic anchoring protein for GABAA receptors and plays a crucial role in regulating GABAergic synapse formation and plasticity. In mice with AD accompanied by anxiety, GABAA receptor function is impaired, and gephyrin expression is reduced; however, DHM reverses these effects (Liang et al., 2014). In addition, DHM reduces anxiety-like behavior in mice by repairing GABAA receptors and synaptic function impaired by social isolation, restoring ATP levels and gephyrin expression (Silva et al., 2020).

DHM also alleviates anxiety by inhibiting neuroinflammation. Social isolation induces hippocampal microglia overactivation and elevates serum corticosterone levels, which, in turn, activate the NF-κB inflammatory signaling pathway in anxiety disorder models. DHM inhibits these changes, reducing anxiety-like behavior in mice (Al Omran et al., 2021). This suggests that anxiety states may be linked to neuroinflammatory responses and that DHM exerts antianxiety effects by modulating the NF-κB pathway and suppressing neuroinflammation.

#### 4.4.2 Role of DHM in the treatment of depression

DHM significantly improves depression-like behavior by inhibiting neuroinflammation and regulating the expression of neurotrophic factors. Studies have demonstrated that DHM attenuates the activity of the AGE-RAGE signaling pathway within the hippocampus of mice, leading to a reduction in the production of proinflammatory cytokines, including TNF-α, IL-6, and IL-1β. This modulation is associated with the manifestation of antidepressant effects (Huang et al., 2022). In the lipopolysaccharide-induced depression model, DHM significantly alleviated depression-like behavior in mice, reduced the expression of the microglial marker CD11b, and dose-dependently inhibited the secretion of inflammatory factors, such as TNF-α, IL-6, IL-1β, COX-2, and inducible nitric oxide synthase (Wei et al., 2022). The antidepressant effect of DHM is also related to the activation of the BDNF/TrkB signaling pathway. In a lipopolysaccharide-induced depression mouse model, DHM exhibited stronger antidepressant effects than those of typical antidepressants. This mechanism may involve the inhibition of neuroinflammation and activation of the ERK1/2–CREB signaling pathway, which promotes GSK-3β phosphorylation and enhances BDNF expression (Ren et al., 2018). BDNF and neuroinflammation are closely associated with depression-like behavior, and BDNF expression is reduced in the hippocampus of patients with major depressive disorder (Chan et al., 2016; Nuernberg et al., 2016).

#### 4.4.3 Multitarget regulatory mechanism of DHM

DHM demonstrates anxiolytic and antidepressant properties through inhibition of the TLR4/Akt/HIF-1α/NLRP3 signaling pathway. This inhibition subsequently leads to a reduction in neuroinflammation and the release of proinflammatory factors (Wei et al., 2022). DHM also promotes neuronal survival and synaptic plasticity by activating the BDNF/TrkB signaling pathway (Ge et al., 2019). In addition, DHM achieves synergistic relief of pain and depression symptoms by downregulating P2X7 receptor expression, blocking the ATP-gated ion channel-mediated ERK1/2 phosphorylation cascade, and reducing TNF-α and IL-1β levels (Silva et al., 2020).

In a diabetic neuropathy model with comorbid depression, DHM alleviated both neuralgia and depressive symptoms by suppressing P2X7 receptor expression, thereby reducing ERK1/2 phosphorylation and proinflammatory cytokines (TNF-α, IL-1β) (Ge et al., 2019; Guan et al., 2019). In addition, DHM restores gephyrin expression, improves inhibitory synaptic transmission efficiency, and reverses GABAA receptor dysfunction in AD-related anxiety (Silva et al., 2020).

In summary, DHM shows considerable potential for the treatment of anxiety and depression. It significantly alleviates symptoms of mental illness by regulating GABAergic neurotransmission, inhibiting neuroinflammation, promoting neurotrophic factor expression, and modulating P2X7 receptor expression through multiple mechanisms. The multitarget regulatory effects of DHM provide a promising molecular intervention strategy for psychiatric disorders (Jia et al., 2019b; Jia et al., 2019a; Le et al., 2016; Shi et al., 2015; Stuart et al., 2013; Zhou et al., 2017). Future studies should investigate the clinical application potential of DHM and offer patients safer and more effective treatment options.

### 4.5 Application in neuralgia

Neuralgia is a common symptom of various neurological disorders, often resulting in significant pain and a diminished quality of life. DHM, a natural compound with diverse biological activities, has demonstrated potential in alleviating neuropathic pain.

#### 4.5.1 DHM alleviates neuropathic pain by regulating microglial polarization

Research has shown that DHM can significantly alleviate neuropathic pain by promoting the phenotypic transition of microglia from the proinflammatory M1 state to the anti-inflammatory M2 state. Specifically, DHM enhances the activity of aldehyde dehydrogenase 2 (ALDH2), thereby promoting the proliferation of M2 phenotype cells and reducing pain sensitivity induced by nerve damage (Zhang et al., 2020a). In a mouse model of neuropathic pain, DHM administration significantly increased the mechanical withdrawal threshold, indicating a marked analgesic effect.

Zhang et al. further demonstrated the potential of DHM as an antineuropathic agent. Their study indicated that DHM upregulates the expression of ALDH2, which facilitates the polarization of BV-2 microglial cells from the M1 to the M2 phenotype. This phenotypic shift contributes to reduced pain hypersensitivity associated with nerve injury. Consequently, ALDH2 has emerged as a promising therapeutic target for the treatment of neuropathic pain (Zhang et al., 2020b).

#### 4.5.2 DHM alleviates neuropathic pain by inhibiting proinflammatory cytokines

In addition, DHM has been shown to suppress the expression of proinflammatory cytokines, including TNF-α and IL-1β, in microglial cells, key mediators in the pathogenesis of neuropathic pain (Guan et al., 2019). By inhibiting the release of these cytokines, DHM exhibits significant efficacy in mitigating neuropathic pain symptoms and improving patients’ quality of life. In a chronic sciatic nerve compression model, DHM intervention resulted in a 58% increase in the mechanical foot contraction threshold. Furthermore, it led to a 40%–65% reduction in TNF-α and IL-1β expression levels in the spinal dorsal horn. These findings confirm that DHM suppresses neuropathic pain signal transduction by inhibiting NLRP3 inflammasome activation (Guan et al., 2019).

#### 4.5.3 Therapeutic potential of DHM in diabetic neuropathy with comorbid depression

Preclinical studies have also demonstrated the therapeutic potential of DHM in managing diabetic neuropathy accompanied by depressive symptoms. DHM improves both neuralgia and depression-like behaviors in diabetic rats by modulating the BDNF/TrkB signaling pathway (Ge et al., 2019). This discovery provides novel insights into the integrated treatment of neuropathic pain and its associated neuropsychiatric complications.

In a streptozotocin-induced diabetic rat model, DHM not only improved mechanical allodynia (with the threshold increasing from 3.2 g to 8.7 g) but also enhanced hippocampal neuronal survival by 32% through upregulation of the BDNF/TrkB pathway. Simultaneously, it alleviated both pain symptoms and depression-like behaviors (Ge et al., 2019).

In summary, DHM demonstrates considerable potential for the treatment of neuropathic pain. Through multiple mechanisms, including modulation of microglial polarization, inhibition of proinflammatory cytokine release, and enhancement of neurotrophic signaling, DHM may offer novel and effective therapeutic options for neuropathic pain and its comorbid conditions.

### 4.6 Application of DHM in other central nervous system diseases

DHM has demonstrated substantial anti-inflammatory and antioxidant properties, rendering it a promising candidate for the treatment of inflammation-related neurological disorders. For instance, research has shown that DHM attenuates the expression of cytokines associated with neuroinflammation by inhibiting NLRP3 inflammasome activation, thereby mitigating neuronal damage (Hong et al., 2025). This mechanism may have clinical significance for multiple sclerosis and other neuroinflammatory diseases, including ischemic brain injury. In the ischemia-reperfusion model, DHM significantly improved the survival rate of nerve cells and reduced oxidative stress and apoptosis by activating the Wnt/β-catenin signaling pathway (Tao et al., 2022). This discovery provides new insights into the neuroprotective effects of DHM following stroke and suggests that it may serve as an effective adjuvant therapeutic agent. In addition, DHM has been investigated for the treatment of diabetes-associated neuropathy. DHM improves blood glucose levels in diabetic mice and alleviates diabetes-induced nerve damage by modulating the AMPK signaling pathway (Wang et al., 2023). DHM has also shown promise in the treatment of hearing loss. It protects auditory cells from aminoglycoside-induced ototoxicity by inhibiting oxidative stress, indicating its potential application in managing auditory disorders (Han et al., 2020). Furthermore, DHM has demonstrated efficacy in inhibiting tumor metastasis. In nasopharyngeal carcinoma cell lines, DHM significantly inhibits cellular migration and invasion, potentially through suppression of MMP-2 expression (Jiang et al., 2025). This antimetastatic property provides an additional perspective on the use of DHM in treating tumor-related neuropathies. Taken together, the application of DHM in various central nervous system diseases shows substantial promise. Its anti-inflammatory, antioxidant, neuroprotective, and antimetastatic effects offer a solid foundation for future clinical research. With continued exploration of its mechanisms of action, the scope of DHM’s application in central nervous system disorders is expected to broaden and become increasingly validated.

### 4.7 Potential applications in other diseases

Beyond the effect of DHM on the central nervous system, additional clinical applications include type 2 diabetes and insulin resistance, nonalcoholic fatty liver disease (NAFLD), hyperlipidemia, oncology, and cardiovascular protection. Indeed, in clinical trials, DHM supplementation significantly improved glycemic control, renal function, and lipid profiles in diabetic patients. It enhances glucose uptake via GLUT1 translocation and AMPK/Akt pathway activation (Chen et al., 2021a; Li et al., 2017; Sun et al., 2021a). DHM reduces hepatic steatosis, oxidative stress, and inflammation by regulating AMPK, Akt, and PPARγ pathways. A double-blind trial confirmed lowered TNF-α, cytokeratin-18, and improved liver enzymes (Chen et al., 2021b; Li et al., 2017; Sun et al., 2021b). It ameliorates high-fat-diet-induced dyslipidemia by modulating Krebs cycle enzymes and lipid metabolism genes (Chen et al., 2021a; Li et al., 2017). In cancer, DHM induces selective ROS-mediated cancer cell death in breast, liver, ovarian, and other cancers. It inhibits proliferation, migration, and invasion (e.g., via miR-21 regulation in cholangiocarcinoma) and promotes apoptosis (Chen et al., 2021c; Chen et al., 2021b; Wang et al., 2022). DHM reduces myocardial infarction damage, chemotherapy-induced cardiotoxicity, and diabetic cardiomyopathy by activating SIRT3/FOXO3a and Nrf2 pathways. It also improves vascular function and reduces fibrosis (Chen et al., 2021b). Finally, DHM stimulates irisin production, mimicking exercise-induced benefits like enhanced glucose tolerance and energy expenditure (Chen et al., 2021a). Although those other possible applications were not covered in detail in the present review, they could share some mechanisms with those of the neurological benefits of DHM. Table 6 summarizes the clinical trials on DHM.

**TABLE 6:** DHM clinical trials.

ID	Study subject	Design	Indication	Dose range	Status	Key findings and objectives
NCT04780268	DHM for alcohol hangover symptoms	RCT (double-blind, placebo-controlled)	Alcohol hangover	300–600 mg/day (single dose)	Completed	Significantly reduced headache, nausea; good safety profile
ChiCTR2000031326	DHM intervention in nonalcoholic fatty liver disease (NAFLD)	Open-label single-arm trial	Metabolic syndrome	250 mg/dose, 2×/day (12 weeks)	Ongoing	Assess liver fat content (MRI-PDFF) and inflammatory markers
NCT05210790	Dose-finding study of DHM for insulin resistance	Dose-escalation phase I trial	Type 2 diabetes	100–800 mg/day (4 weeks)	Recruitment completed	Preliminary dose-dependent improvement in HOMA-IR (unpublished data)
N/A (Industry-sponsored)	Safety evaluation of DHM nanoparticle formulation	Phase I trial (healthy volunteers)	Bioavailability optimization	50–200 mg (IV vs oral)	Unpublished	Nanoparticle group showed 3.5× higher AUC (Patent WO2022156789)

## 5 Conclusion

DHM, a natural compound recognized for its broad pharmacological profile, has attracted considerable attention for its potential use in treating central nervous system disorders. A systematic review of the literature and rigorous research analyses can provide a more comprehensive understanding of DHM’s mechanisms of action and its therapeutic efficacy across various diseases. Still, effective delivery systems remain to be determined (Singh et al., 2024).

The pharmacological potential of DHM has been primarily evident through its antioxidant, anti-inflammatory, and neuroprotective effects. Empirical research has demonstrated that DHM enhances central nervous system function by modulating oxidative stress responses, reducing neuroinflammation, and promoting neuronal survival. These mechanisms establish a strong theoretical basis for therapeutic intervention in neurodegenerative diseases, such as AD and PD. Still, it must be emphasized that there can be important variability among different animal models of the same condition and that many aspects lack clinical validation. Although numerous studies support the efficacy of DHM, variability in research outcomes remains, possibly due to differences in study design, sample selection, dosage, and administration routes.

When evaluating these results, it is necessary to adopt a rigorous scientific perspective and critically assess the strengths and limitations of each study. Some studies may be constrained by small sample sizes or a lack of appropriate control groups. Therefore, future large-scale randomized controlled trials will be essential to validate the efficacy of DHM. Investigation into the effects of various doses and administration routes will provide further evidence to support clinical application.

Additionally, the bioavailability and metabolic pathways of DHM merit further attention. Current studies suggest that DHM metabolites may influence their pharmacological activity; however, the research in this area remains limited. Future investigations should focus on characterizing the *in vivo* metabolic profile of DHM to optimize its clinical utility.

In the treatment of central nervous system disorders, increasing emphasis is being placed on integrated management models in conjunction with pharmacological therapies. As a potential adjuvant, DHM may exert synergistic effects when combined with other therapeutic strategies. Such combinations may enhance overall treatment efficacy while minimizing adverse effects.

Finally, although DHM demonstrates considerable potential for clinical use, further research is necessary to bridge the current knowledge gap. In the rapidly evolving field of medicine, it is anticipated that future studies will elucidate the mechanisms of DHM and support its application in managing central nervous system disorders. Scientifically robust research designs and in-depth exploration of molecular mechanisms will contribute to the development of safer and more effective therapeutic options. These advancements will improve the quality of life for individuals with neurological conditions and facilitate progress across multiple domains of medicine. Continued attention to emerging DHM research is essential for laying the groundwork for future clinical implementation.
